# Incorporating Paid Caregivers Into Medical Education to Enhance Medical Student Exposure to This Essential Workforce

**DOI:** 10.2196/38329

**Published:** 2022-12-09

**Authors:** Samir Kamat, George Danias, Aneesh Agarwal, Sumanth Chennareddy, Joseph Han, Samuel Lee

**Affiliations:** 1 Department of Medical Education Icahn School of Medicine at Mount Sinai New York, NY United States

**Keywords:** medical education, education, student, communication, perspective, medical student, paid caregiver, caregiver, health care model, home-based health care, patient care, health care provider, medical student, student experience, training, care team, integration, clinical decision

## Abstract

The implications of the COVID-19 pandemic underscored the utility of home-based health care due in part to social distancing requirements, curtailment of elective hospital procedures, and patient apprehension of the health care setting. The pandemic particularly accentuated the integral role of paid caregivers (eg, home health aides, personal care attendants, and other home care workers) in caring for patients with chronic health conditions. Given the paradigm shift toward community- and value-based health care models, paid caregivers are likely to play an even greater role as care team members. Despite the increasingly prominent role paid caregivers are assuming in health care, especially for patients who are chronically ill, in our experience as medical students, we have very little exposure to these care team members, with most interactions occurring in brief, chance encounters. Specifically, we advocate for increased medical student exposure to paid caregivers to facilitate their recognition as valuable care team members. We propose to achieve this through (1) classroom-based module learning with live paid caregivers and (2) plain language communication training to enhance reciprocal engagement.

## Introduction

Although the COVID-19 pandemic underscored the critical role of the many frontline workers who support the general public daily, our ambulatory care rotation exposed us to another unacclaimed part of the care team: paid caregivers. Paid caregivers include home health aides, personal care attendants, and other home care workers who care for people with functional impairments at home [1]. This categorization excludes relatives, friends, and spouses, even those with health care experience, who are unpaid caregivers. Paid caregivers provide essential support to various populations, although they primarily care for geriatric and homebound geriatric patients. The number of homebound adults in the last decade has more than doubled. Among adults aged over 70 years, 4.2 million were homebound in 2020, compared to 1.6 million in 2019, likely due to the results of the pandemic; this further highlights the increasing need for paid caregivers to provide support in the home [2]. By characterizing their roles in clinical and community environments and sharing anecdotes from our educational experiences, we hope to make the case that medical education should more deeply expose students and trainees to paid caregivers.

## Our Experiences With Paid Caregivers Underscore Their Integral but Underrecognized Contributions to Patient Care

As a part of our Geriatric Medicine clerkship, we rotated through the Mount Sinai Visiting Doctors program, which provides home-based primary care for homebound patients in New York [3]. Many patients we encountered had functional impairments that limited their quality of life, and many others relied on paid caregivers to support their clinical care. The most striking finding was the extent of paid support; from well-appointed apartments to government-subsidized units, patients of all socioeconomic statuses routinely relied on paid caregiver assistance in the home environment. In one encounter with a patient with a severe disability, the physician relied on the home health aide for information on the patient’s functioning, including getting out of bed, bowel movement regularity, sleep quality, and medication adherence. Although providers are only present for brief periods with their patients, paid caregivers fill the temporal and informational gaps in providing a more complete picture for improved care delivery.

In general, paid caregivers help individuals with activities of daily living (eg, eating and toileting) and instrumental activities of daily living (eg, housekeeping and meal preparation) [4]. Trained paid caregivers with more specialized skills may be able to provide additional assistance for patients with dementia, behavioral health issues, and palliative care needs [4]. One study found that half of community-residing patients with advanced dementia received paid care, with 30% having part-time paid care and 18% having full-time paid care [5]. Although paid caregivers help with daily activities, they also perform a wide variety of other tasks, including maintaining physical living conditions, participating in family dynamics, identifying emergent clinical changes, and assisting with patient self-care of chronic medical issues [1,4]. Notably, the literature suggests that paid caregivers provide significant mental and emotional health support for patients as well [1,4]. For one homebound patient with dementia, beyond providing physical care, it was evident through the paid caregiver’s interactions and attitude toward the patient that they considered the patient to be similar to family. In addition to characterizing the patient’s symptoms, mood, and life events during our visit, the paid caregiver also demonstrated involvement in supporting the patient’s hobbies, interests, and family obligations. During our interview, the patient indicated that they considered the paid caregiver an integral part of their family. The paid caregiver consistently went “above and beyond” by demonstrating interest in the health of other family members and providing emotional support, as well as through smaller actions such as bringing the patient’s favorite foods. This indicated the strength of the patient–paid caregiver relationship and how paid caregivers may have roles that impact broader dimensions of health and well-being. Due to the profound role they play in care, paid caregivers may be able to improve patient outcomes and support high-value care from long-term care funding programs such as Medicaid [1].

## Current Medical Education Lacks Intentional Student Exposure to Paid Caregivers

Although enlightening, our brief experience with paid caregivers in the home only captures a small fraction of the paid caregiver workforce and their services. Overall, 3 million paid caregivers furnish important services in the community for patients with chronic and acute conditions [6]. Considering the substantial role paid caregivers play in the lives and health of patients, as we characterize through our experiences, we strongly believe that medical education should include deliberate and sufficient exposure to these essential care team members.

As part of the paradigm shift toward value-driven interventions for better chronic condition management, several initiatives have successfully incorporated paid caregivers, recognizing their potential to improve health for certain patient populations. For example, the Mount Sinai Hospital Home Care Collaboration Solutions trained home care staff and home care aides to keep track of changes in their patients’ health statuses [4]. Another study from St. John’s Well Child and Family Center presented pilot data showing a 40% improvement in medication adherence and a decrease in patients’ unhealthy days by over 38% (25.3 to 15.6 months) when trained aides did additional chronic care management tasks and were involved with the medical and social care teams [4]. Interventions involving paid caregivers appear to be on an upward trend, particularly as health policy emphasizes community-based care models [1]. Exposure to these initiatives, whether in direct interactions during clinical rotations or through case-study didactic modules, would provide valuable insight for future health professionals into how a paid caregiver may be helpful for a patient and how to best integrate paid caregivers into the health care team.

As a formative stage of training, medical education is uniquely positioned to highlight the increasingly important roles of paid caregivers. Although we were fortunate to get exposure through rotations, more widespread interactions with paid caregivers are needed, potentially through family medicine or dedicated ambulatory care rotations, so that medical trainees can observe and experience the importance of paid caregivers who care for certain patient populations.

## We Propose Classroom-Based Learning and Plain Language Skills Training to Enable Future Physicians to Better Involve Paid Caregivers in Patient Care

Although experience in the clinical environment with a paid caregiver is ideal, such exposure may not always be possible. Therefore, we propose that a preclinical clinical skills module detailing the role and contributions of paid caregivers in patient care is useful if clinical opportunities are limited. Low clinical exposure can be overcome through classroom-based exposure to paid caregivers, in-person or remote video teleconferencing, and communications training, perhaps through standardized patients. Furthermore, the clinical skills module should include communication skills needed for physician–paid caregiver interactions and video simulations. Errors in patient care may arise due to inadequate health literacy among paid caregivers, which can potentially be prevented by improving physician–paid caregiver communication [7]. Thus, the module should teach learners, as future physicians, how to communicate effectively with paid caregivers in plain language. Intentionally developing plain language communication skills will make physicians more accessible and reduce communication barriers with paid caregivers and patients [8]. Additionally, through module-based learning, experienced paid caregivers can voice concerns, questions, and knowledge of the patient’s care to future physicians, creating bilateral communication between providers. Thus, such a module will emphasize paid caregivers’ broad roles outside of just personal care to build a reciprocal clinician–paid caregiver partnership.

As students will undoubtedly come across paid caregivers in their clinical rotations and careers, early recognition of these critical players will contribute to a more comprehensive medical education. Physicians may not appreciate a paid caregiver’s value to the patient [1]. Specific education in medical training on how the paid caregiver can contribute to the patient’s care, presentation of evidenced-based outcomes on how paid caregivers improve patient quality of life, and allowing students to talk to or hear from paid caregivers in the classroom setting will help raise future physician awareness surrounding the presence and critical roles of paid caregivers for certain patient populations. Early exposure to paid caregivers and how to effectively involve them can afford trainees a more holistic approach to patient care in their future clinical practice.
